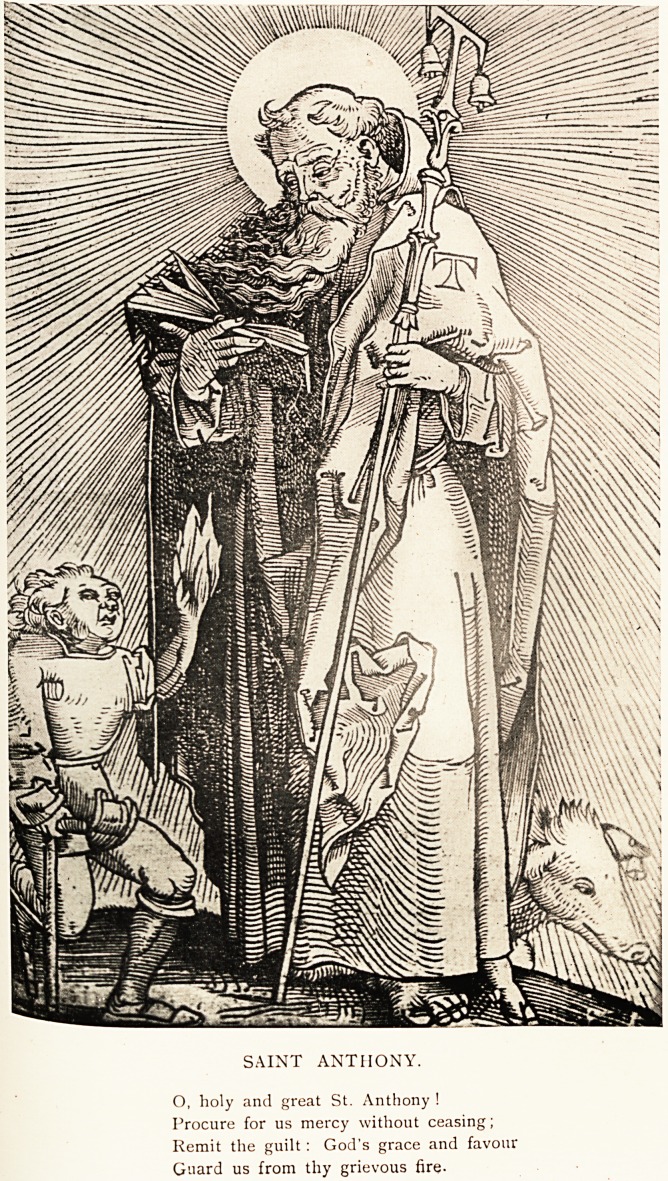# On Some Diseases Bearing Names of Saints

**Published:** 1912-12

**Authors:** Robert Fletcher

**Affiliations:** Late Principal Assistant Librarian, Surgeon-General's Office, Washington, D.C., U.S.A.


### ON SOME DISEASES BEARING NAMES OF SAINTS.

BY

ROBERT FLETCHER, M.D. Columbia and Bristol, M.R.C.S.

*Late Principal Assistant Librarian, Surgeon-General's Office, Washington, D.C., U.S.A.*

---

WHILE the history of medicine has been written with diligence and research in various languages, and in some instances in a portentous number of volumes, there are still many unexplored byways which may lead to discoveries of interest, though they may be of no striking importance. The folk-lore of different peoples, popular superstitions in regard to disease, allusions to medicine in the old chroniclers, poets and dramatists, have contributed something of value to the history of medicine, and to a knowledge of the estimation of the physician in the popular mind. Among these outlying subjects for research the association of diseases with the names of the early saints and martyrs of the Christian Church offers a field which has been but little explored. I have been accustomed for many years to make notes of allusions in this direction, and the quantity thus obtained is really surprising. I have made no search for these references, but may say of them as Falstaff said of Hotspur's revolt—

> "Rebellion lay in his way, and he found it."

A few remarks upon the general subject, with illustrations from some of the more prominent diseases which have been thus provided with names, will, I trust, prove not uninteresting.

The printed works of the hagiographers, such as the *Aurea Legenda*, the *Catalogus Sanctorum* of Peter de Natalibus, Alban Butler's *Lives of the Saints*, the more recent work of Baring-Gould, and above all that monument of pious industry and research, the great Bollandist compilation, the *Acta Sanctorum*, which has now reached to nearly seventy-five folio volumes, contain numberless accounts of the cure of disease by saintly offices. Such features as are merely miraculous form but an incidental part of the subject of this paper. A remarkable dictionary or directory of patron saints (*Die Patronate der Heiligen*), by Dietrich Heinrich Kerler, was published at Ulm in 1905.

From a very early period certain prevalent diseases have been associated with the names of saints who were supposed to take them under their special control. Strictly speaking, it is not correct to apply the title of "Saint" to these holy men and women while recounting their lives or achievements. That title could only apply after canonisation, which necessarily occurred after their death, and in many instances at a very long time, even a hundred years later. Nevertheless, it is convenient to speak of them as they are known in church history or legend. It must not be supposed that prayers and relics were the only curative methods employed by them in treating the diseased. Their simple pharmacopœia provided them with vulneraries and dressings, and as shrines and holy wells became established in reputation, the priests in charge acquired some knowledge of disease and of the modes of treating it. No doubt the fame of these holy places had a semi-miraculous origin, but not infrequently a hospital became in course of time an adjunct to the shrine, and gave a name which has been preserved to this day. We still speak of a lazar-house and a lazaretto, which names are derived from Saint Lazarus, and many an *hôpital Saint-Antoine* preserves the name of the good Saint Anthony.

An interesting feature in the history of saints, and which includes what I may venture to call saints in medicine, is their appearance in art. The early masters devoted a large part of their labours to illustrating religious subjects, and the particular emblem which denoted a saint or martyr was perpetuated with striking fidelity in the numerous paintings which adorned the churches. The subject of many a time-worn painting has through this custom been discovered and identified.

A curious circumstance in the history of saints in medicine is that they were not only believed to cure the diseases associated with their names, but to inflict them also as a punishment. The origin and use of profane oaths, which dates from the earliest period of recorded history, forms an interesting part of the study of popular manners. Not only was the wrath of God invoked upon an enemy, but popular imprecations abounded in calls upon the saints to inflict disease. In Rabelais the Gargantua and the ingenious Panurge frequently pray that Saint Anthony's fire may visit a part of the body not to be mentioned in polite society. Sir Walter Scott, in his poem of "Marmion," makes the impatient Blount address his fellow squire Eustace, who was patiently listening to a long tirade of the Abbess—

> "Saint Anton fire thee! wilt thou stand
> All day, with bonnet in thy hand,
> To hear the lady preach?"

Endless illustrations might be given to show the accepted belief in this double function of the saints to inflict as well as to cure disease. One more must suffice. Barnabee Googe, a poet little known in these days, published in 1570 a translation of the *Regnum Papisticum* of Thomas Neogorgus, under the name of *The Popish Kingdome*. This work is full of curious lore about the saints and their achievements. It is so scarce that but three copies of it are known to exist, only one of which is perfect. It was reprinted, in facsimile of the old English text, in 1880. In speaking of Saint Valentine, who, in addition to his kindly care of lovers in the spring, had especial charge of epilepsy, our poet says—

> "Saint Valentine, beside, to such as doe his power dispise,
> The falling sickness sendes, and helpes the man that to him cries."

To this strange double function may well be applied the saying of Eliphaz to Job, when advising him to put his trust in the mercy of Jehovah—

> "He wounds and heals the wound:
> He strikes and His own hands heal."

Among canonised saints there are forty who are said to have been physicians. Three of the Popes are entitled to the same distinction, namely John XXII, Paul II, and Nicolas V.

When the saint was also a martyr, something connected with his torture or mode of death often determined the form of disease which he was afterwards supposed to control. In art, too, some token of the martyrdom nearly always appears. For example, Saint Agatha of Catanea was cruelly tortured before being put to death. Her breasts were cut off, and she is represented in art as holding in her hand a pair of pincers from which a nipple is protruding. Diseases of the female breast, therefore, fell to her charge, and in course of time she became the patron saint of nursing women. Saint Apollonia was another virgin martyr. Her jaws were broken and her teeth dashed out. She is represented in art as holding a tooth either in her hand or in a pair of pincers. A form of prayer to Saint Apollonia for the cure of toothache is still in use in some parts of France.

Notwithstanding the widespread custom of invoking the aid of the saints, it should be remembered that the Catholic Church does not authorise it. It is expressly declared in the Nuremburg Catechism that to ask their direct aid is pure idolatry, for their intercession with God should alone be asked. This also is declared to be not necessary, but only good and useful.

Defects of disposition as well as bodily diseases were under saintly control. If a child was given to over-crying, was disobedient and sullen, it was vowed to Saint Avertin, and a formulated prayer implored his aid for the young recalcitrant. His name and function are preserved in an old French adjective, *avertineux*, which Cotgrave in his *Dictionary*, published in 1632, defines as " dizzie, giddie, franticke, lunaticke, fantasticall, moodie, humorous, willful, obstinate, stubborne, sullen." The good saint must have been kept busy in family practice. Halliwell gives an old Anglo-Norman word *averty*, meaning "mad, fiery." The word *avertineux* is now obsolete in its original sense, but it is used in veterinary medicine to describe an animal which has the " sturdy or the gid," both of which words describe the staggering movements of a sheep with a cysticercus in the brain. The aid of Saint Avertin was also implored in cases of epilepsy and insanity. I have not met with him in art.

The token of martyrdom in early paintings has sometimes led to a purely commercial use. Saint Blaise, or Blasius, and, in Italian, Biagio, was a bishop of Armenia who suffered martyrdom under Licinius in the year 316. His skin was torn in shreds with a large iron comb, and he is represented in art as holding that instrument in his hand. From this circumstance, the Guild of Woolcombers in the Low Countries adopted him as their patron saint.

Saint Blaise was held to be potent in relieving diseases or obstructions of the throat. It is told that on the way to his martyrdom he extracted a fishbone from the throat of a boy who was at his last gasp, and restored him to life. There is a curious passage which refers to this saint in the works of Aëtius, the ancient Greek physician who flourished about the middle of the sixth century. It reads thus, translated—

"For the relief of those who have swallowed something [which has lodged] in the tonsils, immediately cause the sick one to sit obedient before thee, and say: 'Come forth bone, if bone thou be, or stick, or whatsoever thou mayest be, just as Jesus Christ brought forth Lazarus from the tomb, or as Jonah came out from the whale.' And, taking the sick one by the throat, say: 'Blasius the martyr and servant of Christ saith Either come up or go down.'" The edition from which this extract is made is the Latin translation of Basil, 1535. It is annotated by the learned Cornarius, but his only comment upon this surprising instruction is embodied in a marginal note, *superstitiosum*. There is no doubt that many popular medical superstitions are survivals of some ancient, perhaps very ancient, medical dogma, and this would seem to be a case in proof.

The parcelling out of the human body to the charge of different saints was made the subject of a humorous complaint by Sir John Melton in his *Astrologaster*, published in 1620. He says: "The saints have usurped the place of the zodiacal constellations in the governance of the parts of man's body, and for every limbe they have a saint. Thus, St. Otilia keepes the head instead of Aries; St. Blasius is appointed to govern the necke instead of Taurus; St. Lawrence keepes the backe and shoulders instead of Gemini, Cancer and Leo ; St. Erasmus rules the belly with the entrayles in the place of Libra and Scorpius ; in the stead of Sagittarius, Capricornus, Aquarius and Pisces, the church hath elected St. Burgarde, St. Rochus, St. Quirinus, St. John, and many others which governe the thighes, feet, shinnes, and knees."

This passage is quoted at second-hand, the original work being extremely rare. The only copy of which I have heard is in the British Museum.[1]

Of St. Vitus it is needless to speak, as Hecker has brought together, in his treatise on the dancing mania, all that relates to that saint. St. John shares with St. Vitus the charge of convulsionists, but his vigil and feast-day are marked by a great number of strange observances and credulities. One of these was a cure for congenital hernia, over which the saint was supposed to have control. A young tree was split down through the trunk, care being taken not to sever the roots. A boy, whose name had to be John, passed the afflicted infant through the artificial opening in the tree, and a maid, whose name had to be Mary received it on the other side. During this ceremony the aid of St. John was invoked in a formal prayer. The child was passed three times, and in some places seven times, through the opening. The tree was then bound together, and, if the fissure united and became cicatrised, the cure of the hernia was assured and permanent.[2] A tree with a natural opening from the union of its limbs in growth above answered a like purpose, but could not always be found ; neither could it afford the omen of permanent cure. There seems to be a trace of the doctrine of sympathy in this relation of the ruptured infant to the ruptured tree. Another and more probable explanation is that it was symbolical of a new birth. The infant, who was always naked, was passed with some difficulty through the aperture, and by this second birth, with saintly accompaniments, came into the

[1] Another copy was sold at auction, 1903, in London, to Queritch for £12.

[2] A cleft ash-sapling was presented to the Folk-lore Society of London in 1896. This had been used for the cure of congenital hernia in 1894.

world whole and sound. Passing ruptured children through split trees is mentioned by White in his *Natural History of Selborne.* There is a striking analogy between this custom and a very ancient Aryan ceremonial. In India, the doctrine of the new birth, or regeneration of the body during life from its sins and weaknesses, is still entertained. In practising the rite connected with this belief, a large natural aperture in a rock is selected, and the patient is passed through it in a naked state with many ceremonies. The perforated rock is regarded as a sacred symbol of the Yoni.[1]

Saint John's Day is the Midsummer Day, which in classic times was noted for its pagan rites, many of which were engrafted in Christian observances, for example, in the *Johannisfeuer* of the Germans, mentioned in Goethe's Autobiography, and, even latterly, in Sudermann's remarkable play of that title.

The earliest Christian saint who was regarded as the patron of physicians was certainly Saint Luke. He is mentioned by Saint Paul as "Luke the beloved physician," and this is the only reference to his profession in the New Testament. The *Aurea Legenda,* compiled by Jacobus de Voragine, about 1270, was first printed in 1470. Caxton, the English printer, translated it into English, probably from a French version, and published it in 1483 under the title of *The Golden Legend,* or *Lives of the Saints.* In the life of the evangelist in this collection it is said: "Luke was of the nation of Syria, and Antiochian by art of medicine." This would imply the existence of a school of medicine in Antioch at an early period. Why Saint Luke was held to be the especial saint of the insane does not appear from any of his biographies. In art he is always represented with an ox looking over his shoulder or reposing near him. *The Golden Legend,* in accounting for this, gives a fanciful account of a "quadrate of beasts," containing in one

[1] Thomas Taylor, the Platonist, asserts that the doctrine of regeneration by the gradual triumph of the intellect over the senses, revealed only to the favoured few, is the key to the Eleusinian and Bacchic mysteries, and that this doctrine came originally from India. The symbolical rite just described was intended for the multitude, and not for those within the veil.

of its four corners the face of a man, next of an ox, and lastly of an eagle. The man symbolises Matthew, who wrote of Christ's humanity; the lion stands for Mark, who wrote of the resurrection. "For," says the chronicler, "as some say, the fawns of the lion be as they were dead unto the third day, but by the braying of the lion, they been raised at the third day." John is indicated by the eagle as soaring highest of the four, for he wrote of the divinity of Christ. Luke was figured by the ox. "For," says the writer, "it is a moral beast, and its cloven foot signifies discretion." I have met with other allusions to the ox as a type of morality. Perhaps his neutral condition and mild countenance contributed to this reputation.

The quadrate of beasts is taken from a passage in the Book of the Prophet Ezekiel, which refers to cherubim with four faces.

Professor Adolf Harnack, Professor of Ecclesiastical History at Berlin, published in 1892 a work of which the title (translated) is *Medical Matters in Earliest Church History*.[1] Speaking of Luke, he says that church tradition ascribes to him the gospel which bears his name and the Acts of the Apostles also. Both these books, he continues, have undoubtedly been written by a highly-cultured Greek. . . . It has even been asserted that the preface to the third gospel is formed after the pattern of the preface to the *Materia Medica* of Dioscorides. Professor Harnack quotes the passage, but the resemblance seems to me more fanciful than real. Eusebius in his *Church History* terms Luke "a scientific physician," and also states that he came from Antioch. In the sixth century a painting of the Madonna was discovered in Rome with the name Luke, in Greek, signed to it. With the easy credulity of the age, it was at once inferred that the evangelist, who was personally acquainted with the Virgin Mary, painted the portrait from life. In the Eastern Church, Luke is better known as the patron of painters than as a physician. A judicious little book on the Madonna of Saint Luke has been written by Mrs. Bolton,

[1] See also the same Author's recent work, *Lukas der Arzt*, Leipzig, 1906. Translated by J. R. Wilkinson as *Luke the Physician*, New York, 1907.

the wife of the late erudite bibliographer of chemistry, Dr. Carrington Bolton.

The Faculty of Medicine of the University of Lille, on July 6th, 1893, inaugurated a statue of Saint Luke. It was erected in the garden of the University, facing the building of the Faculty of Medicine. Saint Luke is represented as an old man with thick hair and long beard. In the right hand he holds a pen, and in the left a manuscript. The sculptor was Denonvilliers.[1]

Next to Saint Luke in point of time, but much more generally known as patron-saints of physicians, were the martyrs Cosmas and Damianus. The first is sometimes spoken of as Cosmos or Cosmo. They were the elder brothers of a family of five. The Acts which record their trial and martyrdom are still extant, and are considered to be authentic extracts from the proconsular records, though many miraculous incidents have been interpolated by succeeding writers. The five brothers were summoned before Lysias, the Governor of Ægis in Cilicia, in the reign of Diocletian. Cosmas and Damianus, in reply to the questions of the governor, stated that they were of Arabian origin, and were physicians. They declared also that they and their brothers were Christians. Refusing to worship the statues of the gods, they were tortured and finally beheaded. Their martyrdom took place about the year 297. In the year 530, Pope Felix IV built a church in Rome in their honour, and the remains of the five brothers were buried under the altar. Cosmas and Damian were the patron-saints of the famous ducal family of the Medici.

As late as 1700, the Vienna Medical Society celebrated the feast-day of these saints, September 27th. The ceremonial took place in the Stephanus Church. The Society has in its possession a copper-plate representing the saints as ministering to the sick. Imprints from this plate were distributed among the people on the occasion. The two saints are dressed in furred robes and caps, and have a striking appearance of authority. Hermann Peters, of Nuremburg, in his interesting work on ancient pharmacy, gives a copy of this engraving.

[1] *J. d. sc. méd. de Lille*, 1893, ii. 13, 44.

In a Spanish medical catalogue, published in 1900,[1] there is described an address delivered at the "festival dedicated by the physicians of Bilbao to their patrons, Saint Cosmo and Saint Damian." Bilbao, or Bilboa as we term it, is one of the most ancient cities of Spain, and it is interesting to know that this long-practised celebration is still kept up by its physicians. There is still existing in Barcelona a medical society known as the Academia de los Santos Cosme y Damian.

A remarkable bit of history is connected with the names of these patron-saints of physicians. Sir William Hamilton, the British Ambassador at Naples at the end of the eighteenth century, was a distinguished archæologist, though best remembered through his wife, the famous Lady Hamilton. In 1781 he wrote a letter to Sir Joseph Banks describing some strange phallic ceremonies which annually took place at Isernia, a very ancient town in the kingdom of Naples. A large procession took its way to the old church of Saints Cosmo and Damian, where the rites in question were celebrated. It is one of many instances of early Christian assimilation of pagan customs, and the ancient worship of Osiris or Bacchus was thus perpetuated with the substitution of a saint for a heathen god. How Saint Cosmo, whose authentic history exhibits a man of pure and noble life, became thus degraded it is impossible to say. The Governor of Isernia informed Sir William Hamilton that, in the three days' feast of 1781, over 1,400 flasks of Saint Cosmo's oil were bestowed on the worshippers. Anointing with this miraculous oil was performed by the priests for every kind of ailment, and the flask was presented to enable the sufferer to continue the treatment after leaving the shrine.

Another patron-saint of physicians deserves a passing notice. Saint Pantaleon was a native of Nicomedia, and, from his skill in medicine, was made physician to the Emperor Galerius Maximian. He suffered martyrdom in the year 305. Hecker speaks of fourteen saints to whom churches were dedicated

[1] Martinez Núñez (E. P. Zacarias), *La fe y las ciencias médicas. Discurso pronunciado en la fiesta dedicada por los médicos de Bilbao á sus patronos San Cosme y San Damián.* 1900.

SAINT ANTHONY.

O, holy and great St. Anthony!
Procure for us mercy without ceasing;
Remit the guilt: God's grace and favour
Guard us from thy grievous fire.

between Bamberg and Coburg, and to which thousands resorted annually in pilgrimages. Among them are Pantaleon, Vitus, Blasius and St. Margaretha. The latter was the " Juno Lucina " of pregnant women.

In view of the frequent invasions of Europe by the Oriental plague in the Middle Ages, and the frightful mortality consequent thereon, it is not surprising that the intercession of the saints should have been common on such occasions. Many of them are mentioned by hagiographers as having the dreaded disease under their charge, in particular, St. Sebastian. A notice of two must, however, suffice. Saint Roch, or Rochus, or Rocke,[1] as he is variously designated, seems to have been the saint to whom appeals were most generally made. There are no very authentic records of his life. He lived in the fourteenth century, and it is believed that he devoted himself to the care of the plague-stricken during the Black Death of 1348. When he himself was attacked with the disease he retired to a wood, patiently awaiting death. Miraculous care was taken of him, and a dog brought him daily a loaf of bread. An angel appeared to him in a dream and touched his thigh, meaning probably the groin ; the plague-boil burst, and he recovered. In art he is represented with one thigh and leg bare, to which he points. Sometimes he is accompanied by a dog carrying a loaf of bread in his mouth. While there is much that must be

[1] The name of this saint has been spelt and pronounced in a puzzling variety of ways. It is mostly found as Roch, but sometimes with a final " e," which would imply the pronunciation of Roche. The learned antiquarian, Dr. Samuel Pegge, found him in old chronicles as St. Royke and St. Rooke, and says that there was an ancient festival known as Rock-Monday, held in celebration of the saint. From a like confusion of pronunciation, a proverb familiar to most of us, namely, " As sound as a roach," is quite nonsensical. The roach is a small soft fish such as boys angle for in brooks, and in old dictionaries it is spelt roch, without the " a." The original proverb, which is quite ancient, was no doubt " As sound as a rock." There is an analogous French proverb, " Aussi ferme qu'un roc," " as firm as a rock." Littré, in his *Dictionary*, has "Mal de S. Roch," which he defines as a disease to which the stone-cutters of Paris and Fontainebleau are subject from inhaling the silicious dust produced in their occupation. This is, of course, a mere punning title, but it confirms the pronunciation of Roch.

deemed apocryphal in the histories of these good men, their zeal and self-devotion in the care of the diseased has been generally the source of their reputation. Saint Roch is said to be in high repute among the French Canadians. His day is August 16th. There are many churches dedicated to him, and it was around the Church of Saint Roch in Paris that the last desperate rally of *sans-culottes* was made on the 13 Vendémiaire, year IV, otherwise October 5th, 1795. Carlyle, in picturesque phrase, tells how " the bronze artillery officer, by name Napoleon Bonaparte," sent a terrific " whiff of grapeshot " into their ranks, and " blew the French Revolution into space." The rabbets and plinths of the old church showed the marks of these historic volleys for many years.

Another saint, whose name is associated with the plague, lived several centuries before Saint Roch, and his good deeds are recorded in many authentic biographies, as well as in the Proconsular Acts. This was Saint Cyprian, the celebrated Bishop of Carthage. He lived in the third century. He was a man of noble character and of remarkable ability. His life was spared during the persecution under Decius, though he was banished from the city. Upon the death of that emperor he returned to Carthage. In the year 253 the plague devastated the city, spreading with appalling regularity from house to house. The streets were encumbered with the dead and dying, and none were brave enough to help. Cyprian divided the city into districts, over which he appointed superintendents. He called upon his fellow-Christians to aid him in the pious work, and thousands of them who had been released from slavery at the close of the persecution obeyed his call. It is pathetically related that numbers of those employed in these charitable duties were disfigured by frightful scars received in the prisons and mines to which they had been consigned. Under the bishop's vigorous management the pestilence slowly disappeared. In after times Saint Cyprian's aid was invoked in prayers during periods of similar visitations. Th good bishop's life was crowned with martyrdom, for when the Valerian persecution broke out in 258 he was condemned to death for refusing to worship the heathen gods of Rome. It is told that as his white head fell before the executioner's sword, a universal groan burst forth from the vast multitude of spectators, pagans as well as Christians lamenting the loss of their benefactor. In art he is represented only in his episcopal robe and mitre. Saint Cyprian left many manuscripts, which have been preserved. Altogether, considering the early age in which he lived, he comes to us as an unusually authentic and satisfactory personage.

A bit of preventive medicine for horses was practised on Saint Stephen's Day. Barnabee Googe describes the proceeding with a touch of displeasure at the mingling of the name of the protomartyr with the veterinary art—

> "Then followeth Saint Stephen's Day, whereon doth every man
> His horses jaunt and course abrode, as swiftly as he can,
> Until they doo extreamely sweate, and then they let them blood;
> For this being done upon this day, they say doth do them good,
> And keepes them from all maladies and sicknesse through the yeare,
> As if that Stephen any time tooke charge of horses here."

Insanity was under the charge of many saints, both male and female. There was a certain Saint Dymphna who lived in the seventh century. Her father, an Irish or British king, was so enamoured of her beauty that she fled from him with an old priest named Gerebern, escaping to the Low Countries. The father overtook and slew them both. In the year 1200, a church in her honour was erected at Gheel, the present locality of the interesting colony of the insane. It was determined to build a shrine in commemoration of the martyrdom, and "for the healing of such mental disorders as may have come from base diseases." On Saint Dymphna's feast day, May 15th, 1900, the 1300th celebration of the foundation took place. There is, or was, a painting over the altar representing the saint in a cloud imploring the divine mercy for the lunatics who are grouped around her.

Among the many forms of brutal treatment of these unfortunates, one is mentioned by Dr. Arthur Mitchell in his interesting work on the superstitions in relation to lunacy in the Highlands and Islands of Scotland. There is a loch and islet dedicated to

Saint Maree, whose name is spelt in eight different ways. Insane persons were dragged through the waters of the loch by a rope fastened to the stern of a boat which was rowed around the islet. Dr. Mitchell says that the people firmly believe that it is impossible to drown an insane man "because his gall-bladder has burst." I may add that Sir Thomas Browne classes among his Vulgar Errors the popular belief that the bursting of the gall-bladder of a drowned man on the ninth day causes his body to float.

To Saint Fiacre was attributed the power of healing hemorrhoids, fistulas and ulcers of the rectum. He was the second son of a King of Scotland in the sixth century, but wandered to Normandy, where, at Brusiul, a monastery was afterwards erected by him. A stone which was the usual seat of the saint was believed to possess healing power for the afflicted, who were permitted to sit upon it. Henry V of England, the victor of Agincourt, is said in an old chronicle to have died of the *mal de fiacre*, which has been rendered "fistula" in modern histories. Considering his age, thirty-four, it seems probable that the common camp disease of hemorrhagic dysentery may have been the cause of his death. Old French dictionaries have "Fic-S. Fiacre," and define it as "an excrescence or inflamed scab in the fundament." Martial has endless references to this *ficus* or fig, which he attributes to the prevalent vice of pederasty. There is a less odious connection with this saint's name. The first carriages built for hire in Paris were kept in the Rue Saint-Antoine at an inn in front of which hung the head of Saint Fiacre as a sign, and his name was by degrees applied to the new vehicles, which were known as *fiacres*. The word is not much used now, but I can recollect the *fiacre* as the four-wheeled cab of my student days in Paris.

There is one more saint to be brought to your notice, and he merits especial attention. The Great Saint Anthony, as he is termed to distinguish him from other saints of the same name, belonged to an Egyptian family of distinction. He was born in Egypt in the year 251. His life was written by Saint Athanasius, who was his contemporary, and it is held to be a veracious account. As usual, apocryphal stories have been foisted upon the original history. His interest to us lies in the strange disease which was known in the Middle Ages as Saint Anthony's fire. This became its most common appellation, though it was termed *ignis sacer* in the first chronicles which described it. There are over forty names in different languages by which this formidable disease is known, and Ehlers records that Saint Martial, Saint Benedict and Saint Genevieve were also regarded as its patron-saints. The literature concerning it is very extensive, and widely divergent opinions have been held as to its nature and cause. It has been thought that it was a malignant leprosy, an epidemic syphilis, such as in later years broke out at the siege of Naples, a virulent scarlatina, a herpes esthiomenus, a malignant form of scurvy, a gangrenous erysipelas, and, more generally, a dry gangrene, the result of ergotism.

According to Fuchs, there were twenty-eight epidemics of *feu sacrée* from 857 to 1547. The first reference to the disease appears to be in the Annals of the Convent of Xanten on the Rhine.[1] The first authentically described visitation was that of 945, recorded by contemporary chroniclers, Frodoart, Siegebert and Félibien, who saw the disease. They first termed it *ignis plaga, ignis sacer*. The latter name is found in Virgil, and was employed by Celsus, but it appears to have been a species of epizootic distemper, possibly anthrax. The mediæval disease was characterised by intense burning pains and by gangrene of the extremities, the latter becoming coal-black, and detaching themselves from the body, unless death first supervened. The disease had a slow progress. Numbers of persons were carried from great distances to the tomb of Saint Anthony in Vienne (Dauphiné). None of the authors who described the disease from actual observation confound it with the plague, or speak of inguinal buboes or danger of contagion.

Mézeray, the French historian, has confusedly described the *feu sacrée* the *mal des ardents* and the bubonic plague under

[1] "Annales Xantenses" (in *Pertz. Monumenta*, ii. 230).

the name of the *feu Saint-Antoine*, and Ozanam, Angelade and other epidemiologists have copied his statements.

The Commission of the Royal Academy of Medicine of Paris, who made their admirable report in 1776, carefully distinguished between the *feu sacrée* or St. Anthony's fire and the *mal des ardents* or plague. They distinctly show their belief that the former was the dry gangrene due to ergotism. The current statement that it was brought back from the Crusades is absurd. The first well-described epidemic occurred in 945, and the first Crusade was preached up a hundred and fifty years later. It was prevalent in years in which the winter had been rigorous, the summer moist and rainy; years when the harvest was bad, years of dearth and famine. It never existed epidemically in years of abundance. As a rule the epidemic did not exceed one year in duration, unless the dearth extended through two years. All these conditions were favourable to the production of diseased bread grains. Ergot loses its poisonous qualities after a year.

It is to be remembered that in France, where the disease chiefly prevailed, the period comprised in the tenth and eleventh centuries was one of long feudal anarchy, of revolt of the great nobles, of incursions of the Normans, of the setting out of Crusades, which depopulated the country, and left the cultivation of the soil to women and old men, and of frequent interminable rains, which destroyed or diseased the crops. The terrible suffering of the peasantry during this period is vividly described by Mézeray.

He tells us that the bodies of the dead were dug up to serve as food for the living, children were carried off, and famished wretches, concealed in woods, sprang out on passing travellers and killed them for a like ghastly purpose. Well might the lines of an Elizabethan dramatist apply to this hopeless misery—

> "Devouring famine, plague, and war,
> Each able to undo mankind,
> Death's servile emissaries are!"[1]

The disease broke out in Flanders in 1092, and was called *pestis igniaria* to distinguish it from the *pestis inguinaria*, or

[1] Shirley, *Masque of Cupid and Death.*

plague, and the similarity of nomenclature added to the confusion. When it reached England it was spoken of as Saint Anthony's fire. In one great epidemic in France, Mézeray states that 40,000 persons perished, often after a few hours' illness; that the disease attacked them in *les parties honteuses*, which, in other places, he specifies as the groin. This was evidently bubonic plague.

The latest writer on Saint Anthony's fire is Edvard Ehlers, of Copenhagen, whose work was published in Danish in 1895,[1] and in a French translation in 1902.[2] He is a learned and painstaking man, and the translation of his monograph into a more comfortable language than the Danish has made it the standard source of authority in recent works on the subject, such as the clever booklet *From Ergot to Ernutin*, published by Henry S. Wellcome in 1908. Ehlers has seen much of ergotism and raphania as it still exists in Sweden and Norway and some parts of Russia. Small epidemics, family epidemics as he terms them, still occur in those countries, and there were Russian outbreaks of formidable dimensions at Ekaterinoslav in 1881, Tomsk in 1883, and Poltava in 1888. In Livonia, where the grain is always cleaned and dried, ergotism is unknown. He quotes (from Boucher of Lille) the symptoms observed in an epidemic of 1749–50. The first stage lasted from twelve to twenty-one days. Intense pains were felt in the hands and feet, as if glowing iron was piercing them, and violent cramps attacked the muscles. In the second stage, lasting about ten days, there was torpor of the extremities with a sensation of intense cold. If the limbs were warmed, the fiery pains returned. The third stage, which was long and painful, was that of gangrene and separation. Ehlers is of opinion that some cases of acrodynia, Raynaud's disease and erythromelalgia, observed in Sweden, were due to ergot poisoning. He states also that in chronic ergotism a degeneration of the posterior columns of the spinal cord is found, similar to that which is observed in tabes dorsalis. As an example of the confusion produced by the use

[1] E. Ehlers, *Ignis sacer et Sancti Antonii*. Copenhagen, E. Bojesen, 1895.

[2] *L'ergotisme*. Paris, Masson et Cie, 1902.

of the name Saint Anthony's fire, may be mentioned the account given by Dr. Evaristo Garcia before the Anatomical Society of Paris in 1875. He states that there is a disease known in Colombia as the "Mal de Saint-Antoine." It is regarded as a form of leprosy, is of slow progress, is attended with anæsthesia of the extremities, atrophy of the muscles, fatty degeneration, and contraction of the tendons. Complete absorption of the bones of the hands and feet is a striking symptom of the disease. Charcot, he observes, described a similar absorption of the bones in locomotor ataxy. Garcia himself made an autopsy on a case in Bogotá, and exhibited a foot from it to the Society.

Space permits only the barest outline of the literature of this interesting subject, but I venture to suggest the following conclusions.

1. That the mediæval disease known as Saint Anthony's fire was caused by spurred grain.[1] It was mostly confined to the poorest classes, and occurred under meteorological conditions likely to produce the disease of the cereal crops. That extraordinarily severe burning pains in the extremities and entrails were among its first symptoms, and gave the disease its characteristic names of *feu sacrée*, hellish fire (*ignis infernalis*), etc. That livid coldness of the extremities, succeeded by dry gangrene, followed. That natural separation took place, and in many instances the sufferer recovered. Attempts to remove the gangrened limb artificially are said to have always resulted in death. Finally, that the disease in a much milder form exists to this day in Sweden, Norway, and especially in Russia, from the same cause.

2. That the *mal des ardents* or Persian fire was true bubonic plague. That it is described as attacking the private parts, meaning the groin, and producing buboes. That while often called *feu sacrée*, it has no resemblance to the dry gangrenous disease first described.

3. That Saint Anthony's fire was slow in its progress,

[1] This conclusion was reached as early as 1597 by the Medical Faculty of Marburg.

permitting patients to be carried great distances to shrines and hospitals, and could not have been malignant erysipelas. That a few epidemics have been described mostly in later times, namely in the sixteenth and seventeenth centuries, in which acute inflammation with rapid infiltration of the tissues and moist gangrene occurred, and that these were probably cases of malignant erysipelas. Ehlers mentions an epidemic of this kind which he thinks was properly called erysipelatous.

How Saint Anthony's name became applied to the disease in question is a matter of surmise only. He died six hundred years before the first outbreak occurred. His remains were removed from Constantinople to Dauphiné by the Seigneur de la Mothe-Saint-Didier in the year 1070, and it is probable from the high repute of the saint that his aid was early sought on all occasions. Helyot, in his history of religious orders, gives this account of the origin of the first hospital dedicated to Saint Anthony.[1] A gentleman of Dauphiné, Gaston de Vienne, attributed the recovery of his son from the *feu sacrée* to the fervent prayers addressed by him to Saint Anthony on the young man's behalf. He vowed to devote his future life to the relief of those afflicted with the disease. He founded a hospital at Vienne (Dauphiné) in 1092, which was formally recognised by Pope Urban II on June 28th, 1095. At the same time, the Pope established a religious order under the name of Antonists, or Brothers of Saint Anthony, with their chief seat at Vienne. It was not long before many *maladreries*, as they were termed, were established in various parts of France, Germany and Spain, and one was built in London. From allusions in Rabelais and other writers, it seems that the portals of these hospitals were painted red or of a flame-colour, to attract the eye, it may be supposed, of the stricken pilgrim. The walls of the hospital at Vienne were hung round with dry and blackened limbs which had fallen from the victims of the disease, some of which remained as late as 1702. One of these hospitals at Lyons was known as *Domus contractoria*, a significant reference to the contractions of the tendons, which was one of the most painful symptoms of the

[1] *Histoire des ordres religieux, ii.* 4.

disease. Saint Anthony's fire was sometimes termed *morbus spasmodicus.* Some two hundred years after the foundation of the first hospital, when the disease had greatly diminished, the Antonists were transferred to the rule of St. Augustine.

Dr. Salvatore De Renzi, of Naples, has given very particular accounts of epidemics of *ignis sacer* from the well-known one of 947 down to quite recent times. The title of his work, which was published in 1841, is: *Sul clavismo cangrenoso e sul morbo-convulsivo-epidemico.* The term *clavismus* is not, I believe, in any dictionary, and this is the only instance I have met with of its employment. It is derived from *clavus,* a nail or spike, and evidently refers to the shape and appearance of the ergot or spur on diseased rye. De Renzi's conclusions are that Saint Anthony's fire was the dry gangrene of ergotism, and the *mal des ardents* was bubonic plague.

In art Saint Anthony is represented as of a tall and stately figure. He bears a lofty staff with a cross-piece at the summit, forming the Egyptian cross, known as the *crux commissa,* and sometimes as the *tau* cross, from its resemblance to the Greek letter *tau.* The bells attached to it indicate a hermitage as the home of the saint. The hog which accompanies him has a bell tied round its neck or fastened to one ear. There is nothing said in the early life of Saint Anthony about this curious companion, but it constantly appears in his portraits.[1] "By the hog dear to Saint Anthony," was a popular oath in early English.

The accompanying photograph is reproduced from a plate in the *Feldtbuch der Wundartzney* of Hans von Gerssdorff, called Schylhans, published at Strassburg in 1517. The cripple on the knee-crutch has lost a foot, and is holding up his left hand to implore the aid of the saint. The hand is represented as in flames, symbolical of the disease.

[1] In Paul Heitz's curious *Pestblätter des X V Jahrhunderts* (folio, Strassburg, 1901) there is a representation of St. Anthony with his usual attendant hog, God the Father being visible in a cloud, and a pilgrim in the foreground on his knees, imploring the saint's intercession. On the wall alongside are hung legs and other limbs, as dried *ex votis.*

The legend around the picture, which is not reproduced in the photograph, is a prayer to this effect—

> O, holy and great Saint Anthony!
> Procure for us mercy without ceasing;
> Remit the guilt! God's grace and favour
> Guard us from thy grievous fire.

In the fourth edition of von Gerssdorff's work, issued in 1551, he appears to have become sceptical, for around the same engraving is a verse which may be translated thus—

> O, Anthony, thou holy man,
> To heal the sick why say thou can?
> To God our Lord the honour 's due,
> And not to man—not e'en to you!

The history of the *tau* cross before it became a Christian symbol deserves a brief notice. The Egyptian god Canopus was marked with a *tau*. A Buddhist cross is the *tau*, and it was carved on the bark of the sacred tree of the Druids. *Tau* is also the symbol of Thammuz, the Syrian or Phœnician name of Adonis. According to the classic story, the beautiful youth was permitted after his death to pass six months of the year on upper earth with Aphrodite, and, when the time came for his departure to the realms below, weeping and lamentation followed him, and the stream called by his name, on whose banks he encountered the boar, ran blood-red from Mount Lebanon to the sea. The Prophet Ezekiel speaks of "women weeping for Tammuz," as an idolatrous abomination,[1] and Milton has a noble passage relating to him.[2] Hard-headed persons, who reduce the most poetical myth to a symbolical lesson in cosmogony, assert that Adonis represents the generative power of Nature, his absence below indicating the winter season to be followed by the outburst of spring and summer on his return to earth. The blood-red stream too is only the flood tinge of the red clay banks. The *tau* in Bacchic rites was certainly symbolical of the generative power of Nature. With a ring at the top, it became the *crux ansata*, or handled cross, which may be seen in the hands of many gods in Lepsius's great work on Egypt. So ancient and strange a history has this device on the vestment of a Christian saint.

[1] *Ezekiel*, viii. 14. [2] *Paradise Lost*, i. 446–57.

## Figures and Tables

**Figure f1:**